# Transcriptome Analysis and Knockdown of the Juvenile Hormone Esterase Gene Reveal Abnormal Feeding Behavior in the Sugarcane Giant Borer

**DOI:** 10.3389/fphys.2020.588450

**Published:** 2020-10-28

**Authors:** Daniel D. Noriega, Fabricio B. M. Arraes, José Dijair Antonino, Leonardo L. P. Macedo, Fernando C. A. Fonseca, Roberto C. Togawa, Priscila Grynberg, Maria C. M. Silva, Aldomario S. Negrisoli, Maria F. Grossi-de-Sa

**Affiliations:** ^1^Embrapa Genetic Resources and Biotechnology, Brasília, Brazil; ^2^Department of Cellular Biology, University of Brasília, Brasília, Brazil; ^3^PPG in Genomic Sciences and Biotechnology, Catholic University of Brasília, Brasília, Brazil; ^4^Biotechnology Center, Universidade Federal do Rio Grande do Sul (UFRGS), Porto Alegre, Brazil; ^5^Department of Agronomy/Entomology, Universidade Federal Rural de Pernambuco (UFRPE), Recife, Brazil; ^6^Embrapa Coastal Tablelands, Aracaju, Brazil; ^7^National Institute of Science and Technology (INCT) PlantStress Biotech, Brazilian Agricultural Research Corporation (EMBRAPA), Brasília, Brazil

**Keywords:** *Telchin licus licus*, RNA interference, Lepidoptera, insect gut, RNA-seq

## Abstract

The sugarcane giant borer (SGB), *Telchin licus licus*, is a pest that has strong economic relevance for sugarcane producers. Due to the endophytic behavior of the larva, current methods of management are inefficient. A promising biotechnological management option has been proposed based on RNA interference (RNAi), a process that uses molecules of double-stranded RNA (dsRNA) to specifically knock down essential genes and reduce insect survival. The selection of suitable target genes is often supported by omic sciences. Studies have shown that genes related to feeding adaptation processes are good candidates to be targeted by RNAi for pest management. Among those genes, esterases are highlighted because of their impact on insect development. In this study, the objective was to evaluate the transcriptome responses of the SGB’s gut in order to provide curated data of genes that could be used for pest management by RNAi in future studies. Further, we validated the function of an esterase-coding gene and its potential as a target for RNAi-based control. We sequenced the gut transcriptome of SGB larvae by Illumina HiSeq and evaluated its gene expression profiles in response to different diets (sugarcane stalk and artificial diet). We obtained differentially expressed genes (DEGs) involved in detoxification, digestion, and transport, which suggest a generalist mechanism of adaptation in SGB larvae. Among the DEGs, was identified and characterized a candidate juvenile hormone esterase gene (*Tljhe*). We knocked down the *Tljhe* gene by oral delivery of dsRNA molecules and evaluated gene expression in the gut. The survival and nutritional parameters of the larvae were measured along the developmental cycle of treated insects. We found that the gene *Tljhe* acts as a regulator of feeding behavior. The knockdown of *Tljhe* triggered a forced starvation state in late larval instars that significantly reduced the fitness of the larvae. However, the mechanism of action of this gene remains unclear, and the correlation between the expression of *Tljhe* and the levels of juvenile hormone (JH) metabolites in the hemolymph of the SGB must be assessed in future research.

## Introduction

The sugarcane giant borer (SGB), *Telchin licus licus* (Drury, 1773; Lepidoptera: Castniidae), is a devastating pest of sugarcane that has been detected in various countries in South America and Central America (Mendonça et al., 1996). In Brazil, the major producer of sugarcane worldwide, infestations by *T. l. licus* generate significant losses in sugarcane crops, mainly in the northeastern region (Goebel and Sallam, 2011) and with some reports in the state of São Paulo, the main area of cultivated sugarcane (Nicola et al., 2008; Silva-Brandão et al., 2013). The *T. l. licus* caterpillar is a stem borer larva that can reach a longitude of 5–6 cm and displays an endophytic lifestyle. Thus, chemical and biological control methods are inefficient (Negrisoli et al., 2015). Furthermore, to date, there is no established rearing system for *T. l. licus*, and little is known about the physiology and genetics of this insect. Biotechnology approaches, such as RNA interference (RNAi; Niu et al., 2018) and *Bacillus thuringiensis* (*Bt*) toxins (Craveiro et al., 2010), exhibit potential as sustainable control methods, since their mechanisms of action exploit pest physiology and genetics with high specificity.

Pest management by RNAi is an interesting approach for developing entomotoxic molecules with higher specificity and biosafety than current pesticides. The process of RNAi is highly conserved across eukaryotes and is widely used to describe gene function in different species (Huvenne and Smagghe, 2010). To control insect pests, the most commonly used mechanism is based on the exogenous administration of double-stranded RNA (dsRNA) molecules targeting a specific gene that leads to deleterious or lethal phenotypes (Mamta and Rajam, 2017). Some challenges need to be overcome to apply RNAi for pest management in practice efficiently. Stable delivery of molecules in the field, efficient uptake by insects, and the discovery of target genes are the three main objectives to achieve (Niu et al., 2018). In insect pests of the order Lepidoptera, these challenges are especially difficult, as the RNAi response in lepidopterans is highly variable and frequently poor (Terenius et al., 2011; Shukla et al., 2016; Yoon et al., 2017). An accurate choice of target genes, followed by precise validation of their potential for pest management, is often critical for developing RNAi molecules against lepidopteran pests. The use of high-throughput omic technologies is a reliable and robust strategy for screening a large number of target genes in insects (Van Emon, 2015). Transcriptomic data obtained by RNA-Seq are especially valuable because they enable the identification of a target pest’s global gene expression profile in response to any external stimulus or physiological condition. Prior research has shown that transcriptome variations in insects, triggered by their hosts and dietary habits, are a useful source of candidate genes for RNAi and other biotechnological tools (Vogel et al., 2014).

In phytophagous insects, dietary habits and interactions with hosts are determining factors for the survival of insects. Although most adaptations are in response to plant defenses, an efficient exploit of nutritional resources is also required (Schoonhoven et al., 2005). In contrast to their natural hosts, artificial diets used in laboratory rearing provide a nutrient-rich and toxin-free environment for insects’ optimal development (Parra, 2012). Nevertheless, in some species like the SGB, obtain an efficient artificial diet is very challenging (Negrisoli et al., 2015). Some studies, supported by transcriptomic data, have presented candidate metabolic pathways and genes involved with the adaptation processes triggered by natural and artificial diets. These studies’ findings are useful to identify adaptation mechanisms to different diets but are also a source of genes with potential uses in biotechnology-based pest management (Roy et al., 2016; Zhang et al., 2019; Cantón and Bonning, 2020).

The esterase family is a group of enzymes that are highly related to the adaptation processes previously mentioned. These enzymes are encoded by a multigenic family of genes known to be involved in insect development and insecticides resistance. Esterases act by hydrolyzing esters substrates into acid or alcohol groups (Oakeshott et al., 2010). Knockdown of esterase genes, especially those involved in juvenile hormone (JH) metabolism, has been highly effective in reducing the survival of insect pests and, in some cases, has shown promising results in providing RNAi targets for pest management (Kontogiannatos et al., 2013; Chikate et al., 2016; Wang et al., 2016; Vatanparast et al., 2017; Grover et al., 2019).

The JH is a sesquiterpene produced only by insects and crustaceans. Together with ecdysteroids, JH regulates essential processes, such as development, metamorphosis, reproduction, and even behavior. The most abundant isomer in insects is JH-III. Strong titer regulation of this compound in the hemolymph is required for normal insect development and metamorphosis (Goodman and Cusson, 2012). A complex and specific set of enzymes controls the JH titers in insects, from which three enzymes have been identified and characterized as JH-degrading enzymes (Goodman and Cusson, 2012). From these enzymes, juvenile hormone esterase (JHE) has been heavily researched for biotechnological purposes. All these studies have been conducted because the role of JHE is especially important in later larval instars of many lepidopterans and has a large impact on metamorphosis (Kamita and Hammock, 2010; Kontogiannatos et al., 2013; Duan et al., 2016; Zhang et al., 2017).

Studying the gene expression of SGB in response to diet is a useful approach to identify genes and metabolic routes involved in nutritional processes required to achieve an efficient adaptation to new diets (Vogel et al., 2014; Roy et al., 2016; Noriega et al., 2020). Our objective was to identify transcriptomic responses, which are essential for the development of the SGB’s larvae in different diets. Further, we selected one of the identified genes and validated its function and potential as target for RNAi-based control. In this study, we sequenced the gut transcriptome of *T. l. licus* by Illumina sequencing, which produced more and larger fragments than the currently available transcriptome for this insect (de Assis Fonseca et al., 2015). We compared the gut transcriptomes of larvae feed on sugarcane and larvae feed on artificial diet to elucidate the expression features related to diet adaptation. Finally, the function of a candidate juvenile hormone esterase gene (*jhe*) was validated by RNAi. Our findings suggest that the expression of *jhe* in the gut of *T. l. licus* has an important role in the larva’s feeding behavior. By modulating a starvation-like process, the precocious decrease of *jhe* expression leads to larvae malformations and truncated development, resulting in high mortality. Thus, we consider this gene as a promising target for SGB’s management.

## Materials and Methods

### RNA Sample Preparation and Sequencing

Neonates from *T. l. licus* were obtained from infested sugarcane stalks in Maceio, AL (Brazil). Insects were divided into two groups: one group was feed on sugarcane stalk and the other was feed on artificial diet (Supplementary Table S1). Four days after the second molt, third instar larvae with the same body size and weight for each treatment group were separated. Then, guts from 20 treated larvae per sample were isolated and frozen in liquid nitrogen following the protocol of Noriega et al. (2020). Then, total RNA was extracted using a TRIzol reagent kit (Invitrogen, CA, United States). Additionally, an equimolar pool of each sample was included as an extra sample to be used as a reference in the assembly process. The quality of the RNA was assessed with a 2100 Bioanalyzer Instrument (Agilent, CA, United States). Quantification of RNA was performed by using the Qubit RNA BR Assay Kit (Invitrogen, CA, United States). Samples were sequenced twice for each treatment group using the Illumina HiSeq 2000 platform (Illinois, United States).

### *De novo* Assembly and Functional Annotation

Adaptors were clipped, and low-quality sequences were removed from raw data using Trimmomatic v0.33 (Bolger et al., 2014). Next, reads were assembled into contigs using Trinity v2.0.6 (Grabherr et al., 2011), and total contigs were blasted against the National Center for Biotechnology Information (NCBI) nonredundant protein (NR) database using the Blastx algorithm with an e-value cutoff <1E-5. Since the microbiota present in the gut is abundant, contigs that only presented hits with microorganisms or plants were separated into other assembly files and were not used in downstream analysis. Functional annotation was performed using Blast2GO BASIC v4.1 (Conesa et al., 2005), searching protein domains against the InterPro database (Zdobnov and Apweiler, 2001), GO terms (GOs) from the GO database (Harris et al., 2004) and metabolic pathways from the Kyoto Encyclopedia of Genes and Genomes (KEGG) database (Kanehisa and Goto, 2000). The completeness of our transcriptome was assessed by BUSCO (Simão et al., 2015).

### Digital Differential Expression Analysis

To assess expression differences between the two treatment groups (sugarcane-fed and artificial diet-fed larvae), clean reads of each cDNA library were mapped to our assembly using RSEM software (Li and Dewey, 2011). Transcript abundance estimation was performed using the trinity package, and expression levels were calculated based on the fragments per kilobase per million of reads (FPKM) normalization method using the edgeR package (Robinson et al., 2010). Replicate quality was assessed by determining the Pearson correlation between the abundance values of each replicated cDNA library. The significance criteria of DEGs were a false discovery rate (FDR) less than 0.01 and Log_2_ (Fold change) > 2. Finally, GO enrichment analysis was performed using FUNC and REVIGO software (Prüfer et al., 2007; Supek et al., 2011). Visualization of expression profiles was performed using HeatMapper and ClustVis (Metsalu and Vilo, 2015; Babicki et al., 2016).

### Phylogenetic Analysis

Multiple alignment of full-length amino acid sequences of the SGB’s esterases was performed following the classification suggested by Oakeshott et al. (2010). The software used for alignment was MAFFT v7 (Katoh and Standley, 2013). The phylogenetic tree was constructed by RAxML v8 (Stamatakis, 2014) using the maximum likelihood method with the WAG model and 1000 bootstrap replicates.

### RNAi Experiments

Once identified in the transcriptome, a fragment of 349 bp from the gene encoding a juvenile hormone esterase (*Tljhe*) was cloned by polymerase chain reaction (PCR) using the following primers containing the T7 promoter sequence: forward (5'-3'): TAATACGACTCACTATAGGGAGAGTATTTCGCTGGGAGTCTGAG and reverse (5'-3'): TAATACGACTCACTATAGGGAGA GTCGTCATCCAGTCCTTCATT. The template for PCR was cDNA obtained from RNA samples of gut tissue. To produce dsRNA molecules (dsJHE), the PCR product was used as template for *in vitro* transcription and purification using the MEGAscript T7 Kit (Invitrogen) following the manufacturer’s instructions. Prior to validating dsRNA molecules, the expression of *Tljhe* was evaluated by comparing the gut and carcass of three late developmental stages of the SGB (fifth instar larva or L5, sixth instar larva or L6, and prepupa) and the entire body of the pupa. Then, two different methodologies were employed to deliver the dsRNA molecules into fifth- and sixth-instar larvae of the SGB: microinjection directly into the hemocoel and feeding with diet pieces containing dsRNA in aqueous solution. A Gastight Micro Syringe Luer 1701LT (10 μl) (Hamilton Co. NV, United States) with a needle of 51 mm, gauge 26S (Allcrom, São Paulo, Brazil) was used to microinject various amounts of dsRNA (1–10 μg) with a constant volume of 4 μl. Diet delivery was performed by feeding the larvae with 4-cm^3^ cubes of artificial diet containing 10–50 μg of dsRNA. The dsRNA molecules were applied to the surface of each cube and left to dry for 30 min at room temperature. RNase-free water and dsRNA molecules targeting a 300-bp fragment of the gene encoding green fluorescent protein (dsGFP) were used as controls. Experiments were performed in triplicate using five individuals each time.

### Effect of dsRNA Molecules on SGB Development

An efficient amount of dsJHE (25 μg) was determined and delivered to fifth- and sixth-instar larvae following the diet-feeding methodology. The same amounts of dsGFP were used as controls, and treated larvae were previously starved for 24 h. After an initial delivery of 25 μg of dsRNA in time zero, the diet containing dsRNA molecules was renewed three times every 48 h (25 μg of dsRNA each time for a total of 100 μg). After 8 days, 20 g of fresh diet was provided to treated larvae, and development was observed up to pupation (30 days for L5 larvae and 15 days for L6 larvae). The morphology of individuals was recorded at different times using a Leica DFC310 FX digital camera coupled to a Leica MZ12.5 Stereomicroscope (Leica Microsystems, Wetzlar, Germany). Furthermore, nutritional effects and feeding rate were evaluated on treated larvae. To this end, larval weight was measured each day during the experimental period, and net diet intake (NDI) was calculated at three different points (10, 20, and 30 days after dsRNA delivery) for L5-treated individuals and at the end of the experimental period (15 days) for L6-treated larvae. Experiments were performed in triplicate using 30 individuals each time.

### Off-Target Sequences Analysis

To evaluate the specificity of our designed dsJHE molecule, we performed a blastn analysis using as query the DNA sequence of *Tljhe* (contig 44486c1g1i2) to the nr (non-redundant) databases of the NCBI. We selected a model species for each order of the Superorder Endopterygota (Holometabolous insects). The species *Drosophila melanogaster*, *Apis mellifera*, *Bombyx mori*, and *Dendroctonus ponderosae* were selected for the comparison. Additionally, we used the software siFi21 (Lück et al., 2019) to identify suitable small-interfering RNAs (siRNAs) in the sequences obtained from off-target species. A length of 19–21 nucleotides and 0–2 mismatches were used as parameters in the analysis.

### Reverse Transcription Quantitative Real-Time PCR

For all expression analyses, total RNA was extracted from a pool of five treated larvae using the TRIzol reagent kit (Invitrogen, CA, United States), and RNA from the gut and carcass was extracted separately. Total RNA was treated with DNase I (Invitrogen, CA, United States), and synthesis of first-strand cDNA was performed starting from 2 μg of RNA samples using Oligo(dT) 30 primer and M-MLV Reverse Transcriptase (Invitrogen) following the manufacturer’s instructions. The reverse transcription quantitative real-time PCR (RT-qPCR) reactions were performed in a CFX96 Touch™ Real-Time PCR Detection System (Bio-Rad, CA, United States) using the SYBR™ Green system (Promega, WI, United States). Each reaction was performed with 2 μl of diluted cDNA (1/20), 0.5 μM of primer pair, and 5.0 μl of SYBR™ Green in a total volume of 10 μl. In each run, an initial step of 95°C for 15 min was followed by 40 cycles of 95°C for 30 s and 60°C for 60 s. All samples were evaluated in three biological replicates with three technical replicates each. The Cq values were recalculated using Real-time PCR Miner software,^1^ as well as the amplification efficiencies of each pair of primers. Expression analysis was performed following the Pfaffl method (Pfaffl, 2001) using qbase+ software (Biogazelle, Gent, Belgium). To select suitable reference genes, reference gene stability analysis was performed using the entire set of samples in our experiments with the following genes: β-actin (*β-act*), α-tubulin (*α-tub*), glyceraldehyde 3-phosphate dehydrogenase (*gapdh*), translation elongation factor 1 alpha (*ef-1α*), ubiquitin (*ubq*), phosphoribosyl pyrophosphate synthetase (*prpp*), and ribosomal subunit 18S (*rps18*). Reference genes selected were *gapdh* and *rps18*. A list with primer specifications is provided in Supplementary Table S2.

### Statistical Analysis

For statistical analysis of RT-qPCR data, expression values were transformed using Log10 to accomplish homoscedasticity and normality assumptions. Then, one-way ANOVA followed by Tukey’s *post hoc* test was performed by using the software IBM SPSS Statistic v25®. All error bars are the means ± SD. *p* < 0.05 was used to determine significance in all cases. The survival analysis was performed using the Mantel Cox test to compare survival distribution and Kaplan-Meier testing results to estimate the probability of survival for each treatment. The weight loss was evaluated by using a generalized additive model (GAM). The day in which the linear regression pattern changes (day *Z*) was determined for the model, and the regression coefficients were calculated for each treatment before and after the day *Z*. Then an *F* test with Bonferroni correction was used to compare coefficients between treatments. Differences in the diet consumption rate were evaluated by a one-way ANOVA followed by Tukey’s *post hoc* test. All bar and scatter plot figures were generated using SigmaPlot v12.0 (Systat Software Inc.).

## Results

### Sequencing, Assembly, and Annotation

A total of 131.2 million paired-end (PE) reads were sequenced from cDNA libraries of *T. l. licus*, of which 113.8 million (86.7%) were high-quality reads. Then, a *de novo* assembly containing 121,538 contigs was generated, of which 54,162 (44.6%) sequences had at least one putative homolog identified in the NR database. Additionally, 1,054 contigs showing higher similarity with microorganisms were not included in the downstream analysis. The completeness of this transcriptome was 98% according to BUSCO metrics (Supplementary Table S3). A bioproject containing all raw data files is available in GenBank (NCBI) under the accession PRJNA645418.

Most hits in BLAST analysis to the NR database were to the lepidopteran species *Papilio xuthus* (18.3%), *Papilio machaon* (13.6%), and *Amyelois transitella* (12.9%). Additionally, local BLAST to the available transcriptome of the SGB yielded a total of 34,200 (64.4%) hits in our transcriptome from 16,357 (68.7%) contigs of the current published study (de Assis Fonseca et al., 2015). Detailed results of BLAST analysis are shown in Supplementary Figure S1.

Functional annotation of the SGB transcriptome allowed us to identify 38,498 contigs (72.5%) associated with at least a protein family from the InterPro database (Supplementary Figure S2A), with Cytochrome P450 (IPR001128) serving as the family with most annotations (300). Additionally, 2,666 GO terms, distributed among 26,775 contigs (16,521 unigenes), were found in the gene ontology annotation (Supplementary Figure S2B). Finally, 2,907 sequences were successfully classified into 117 metabolic pathways following KEGG annotation. More heavily represented pathways were carbohydrate and amino acid metabolism, while xenobiotic metabolism and biosynthesis of antibiotics appeared in the top five annotated pathways (Supplementary Figure S2C).

### Differential Expression and GO Enrichment Analysis

**Figure 1:**
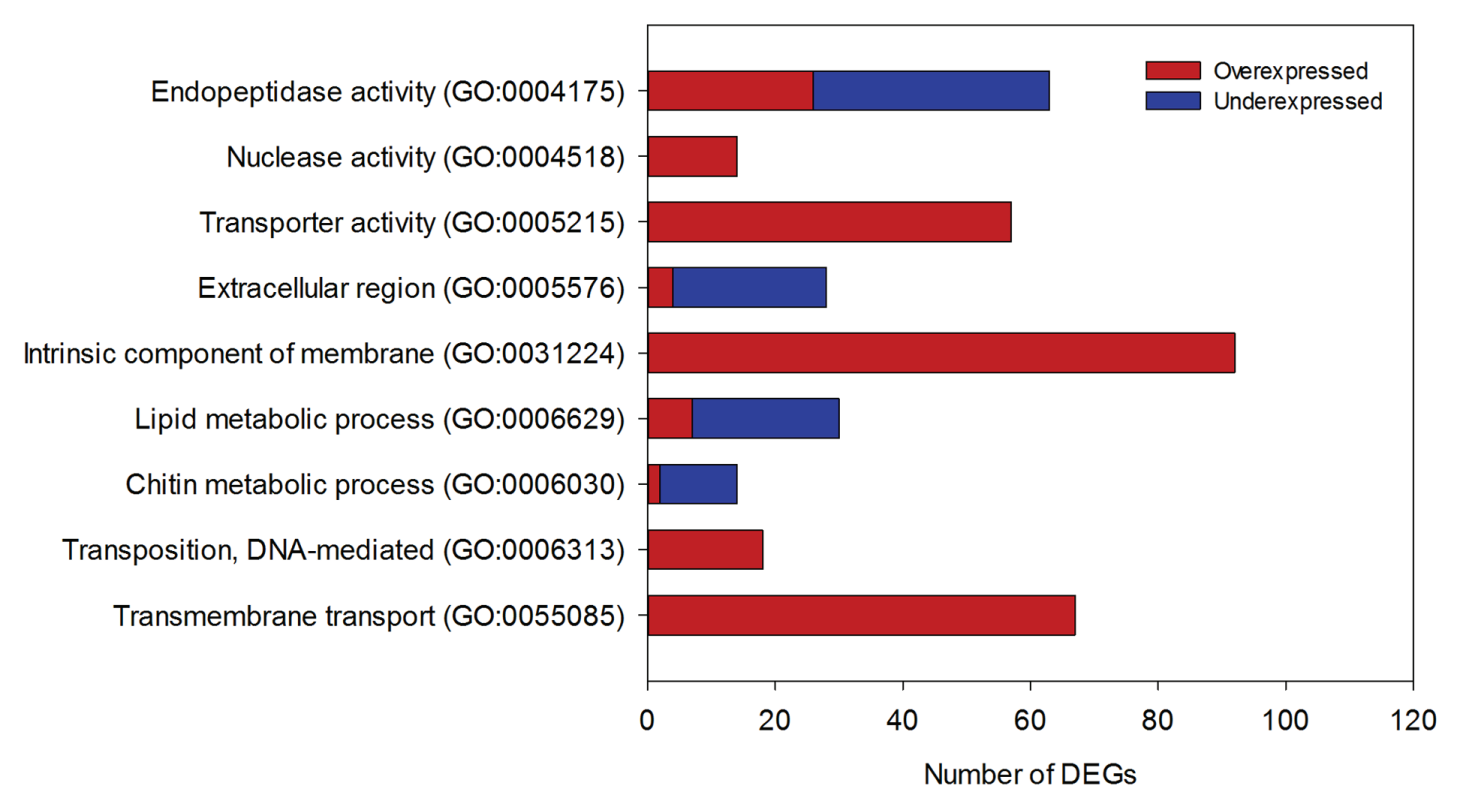
Distribution of differentially expressed genes (DEGs) among the gene ontology (GO) terms with the highest level in the gene ontology hierarchy. Color bars represent the pattern of gene expression in sugarcane-fed larvae compared to artificial diet-fed larvae. *y*-axis: GO Terms. DEGs, differentially expressed genes.

Prior to evaluating expression differences between artificial diet-fed larvae and sugarcane-fed larvae, a total of 84.5% of clean reads were successfully mapped against our assembly. Next, differential expression analysis (DEA) was performed using artificial diet-fed conditions as a reference. Overall, 3,993 contigs were identified as differentially expressed genes (DEGs), of which 2,074 and 1,919 were overexpressed and underexpressed in sugarcane, respectively. From the DEGs identified, 1,302 (32.6%) were transcripts without any match against the NR protein database of the NCBI (NA transcripts; Supplementary Figure S3). To provide a functional view of the transcriptional regulation of SGB larvae in response to diet, GO enrichment was performed separately for each DEG dataset. After filtering redundancies, 57 terms were enriched (full list provided in Supplementary Table S4), of which GO terms in the highest level of ontological hierarchy and related to catalytic processes, membrane proteins, and transport are highlighted in Figure 1.

### Effect of Diet on Nutrition, Detoxification, and Hormonal Regulation

**Figure 2:**
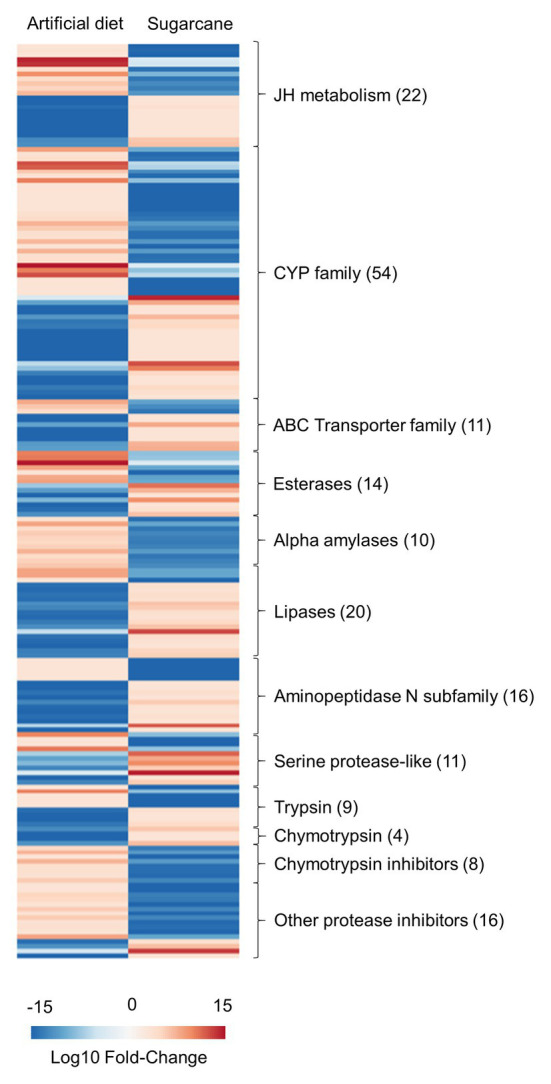
Heatmap showing the expression profile of transcripts clustered into functional groups from highlighted physiological processes. The number within parentheses represents the number of DEGs. Statistical cutoff correspond to a false discovery rate (FDR) less than 0.01 and a Log_2_ (Fold change) > 2. Fold-change is based on fragments per kilobase per million of reads (FPKM) values.

We highlighted some DEGs and clustered them into functional groups and protein families involved in the major physiological processes observed in our analysis (Figure 2). Genes associated with detoxification processes, such as those belonging to cytochrome P450 (CYP) and ATP-binding cassette (ABC) transporter families, presented overexpression of genes in both conditions. Additionally, DEGs related to JH metabolism showed similar patterns. Otherwise, some families involved with digestion showed a more specific pattern, such as alpha amylases, which were only overexpressed in the diet-fed larvae. Additionally, most of the transcripts encoding proteases and lipases were overexpressed in larvae fed sugarcane. Interestingly, the presence of overexpressed genes encoding protease inhibitors was abundant in larvae feed on artificial diet.

### Esterase Gene Distribution in the SGB Gut Transcriptome

**Figure 3:**
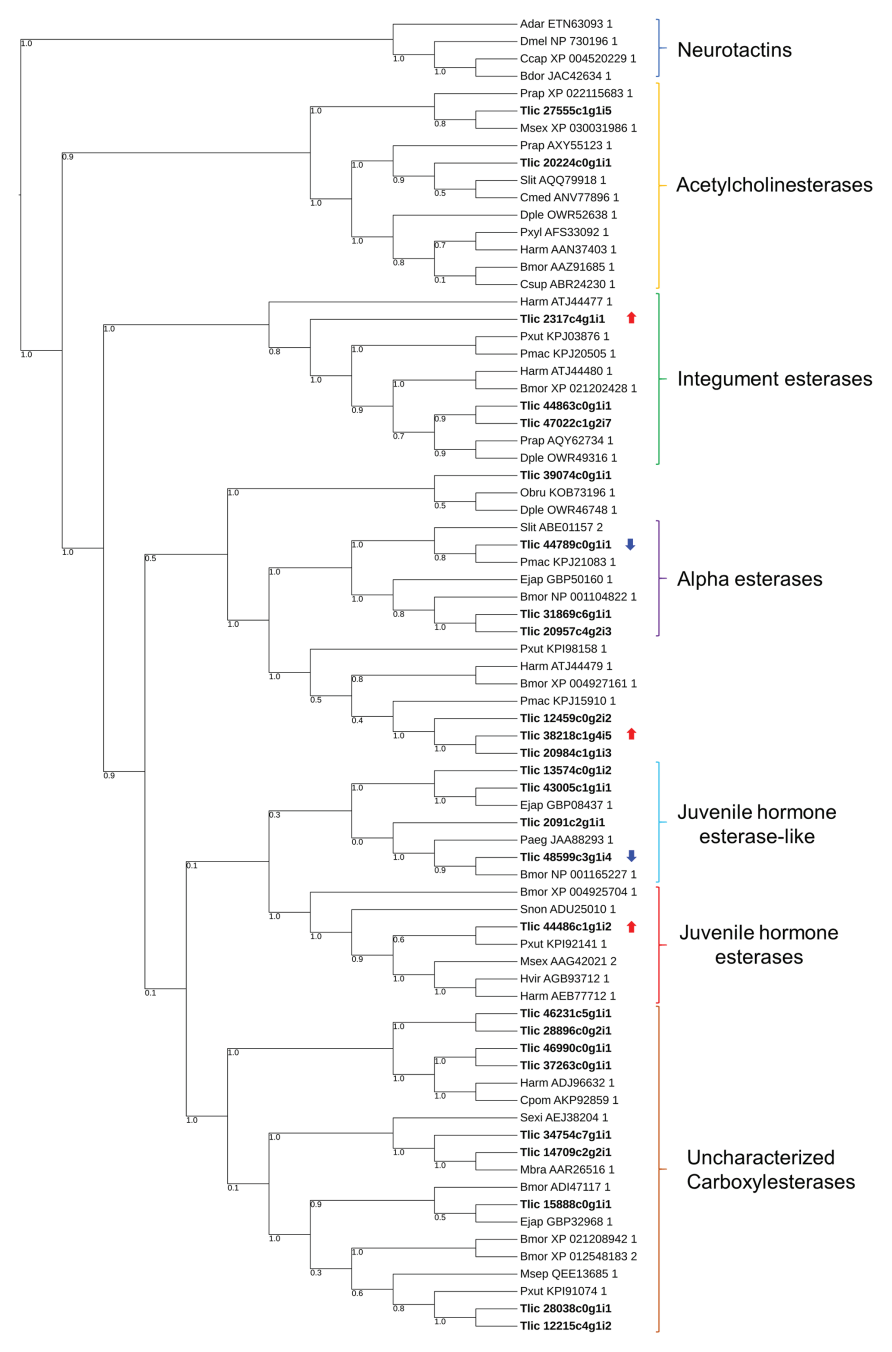
Phylogenetic tree of lepidopteran esterases. Numbers shown in the tree branches represent bootstrap values (only values over 0.7 are given). Identifiers denoted with bold letters represent *T. l. licus* sequences. Arrows represent DEGs overexpressed (red) and underexpressed (blue) in sugarcane-fed larvae. Accession numbers (NCBI) are given next to the species abbreviation. Adar, *Anopheles darlingi*; Dmel, *D. melanogaster*; Ccap, *Ceratitis capitata*; Bdor, *Bactrocera dorsalis*; Prap, *Pieris rapae*; Msex, *Manduca sexta*; Slit, Spodoptera litura; Cmed, *Cnaphalocrocis medinalis*; Dple, *Danaus plexippus plexippus*; Pxyl, *Plutella xylostella*; Harm, *Helicoverpa armigera*; Bmor, *Bombyx mori*; Csup, *Chilo suppressalis*; Pxut, *P. xuthus*; Pmac, *P. machaon*; Obru, *Operophtera brumata*; Ejap, *Eumeta japonica*; Paeg, *Pararge aegeria*; Snon, *Sesamia nonagrioides*; Hvir, *Heliothis viriplaca*; Cpom, *Cydia pomonella*; Sexi, *Spodoptera exigua*; Mbra, *Mamestra brassicae*; Msep, *Mythimna separate*. The tree was constructed using the maximum likelihood method with the WAG model, and 1000 bootstrap replicates.

In total, we classified 22 transcripts with full-length open reading frames (ORFs) into six different groups of esterases and classified four transcripts into uncharacterized groups based on the phylogenies proposed by Oakeshott et al. (2010) and Tsubota and Shiotsuki (2010). Additionally, 14 transcripts among the DEGs were annotated as esterase-like, although only five contained full-length ORFs and were included in our phylogeny (Figure 3). According to our phylogeny, only one transcript corresponded to a juvenile hormone esterase gene (*Tljhe*). We identified a transcript of 2,719 bp containing a full-length ORF of 1,710 bp encoding a protein with 570 amino acid residues (Supplementary Figure S4). In our sequence, we identified all five motifs conserved in JHEs according to Wogulis et al. (2006), as well as the residues forming the catalytic triad.

### RNA-seq Data Validation by Real-Time Quantitative Reverse Transcription PCR

The reference genes selected were *gapdh* and *rps18* based on their stability value given by geNorm analysis (Supplementary Table S5). The expression of DEGs encoding predicted esterases with full-length ORFs was evaluated by RT-qPCR, and the Pearson correlation of results obtained by this technique and the expression obtained from RNA-seq was 0.99 (Supplementary Figure S5). The highest expression differences were observed for the gene *Tljhe*, which was overexpressed in larvae feed on sugarcane thatch (12.8-fold in RNA-seq and 4.4-fold RT-qPCR).

### Knockdown of *Tljhe* in Different Larval Instars and Tissues

**Figure 4:**
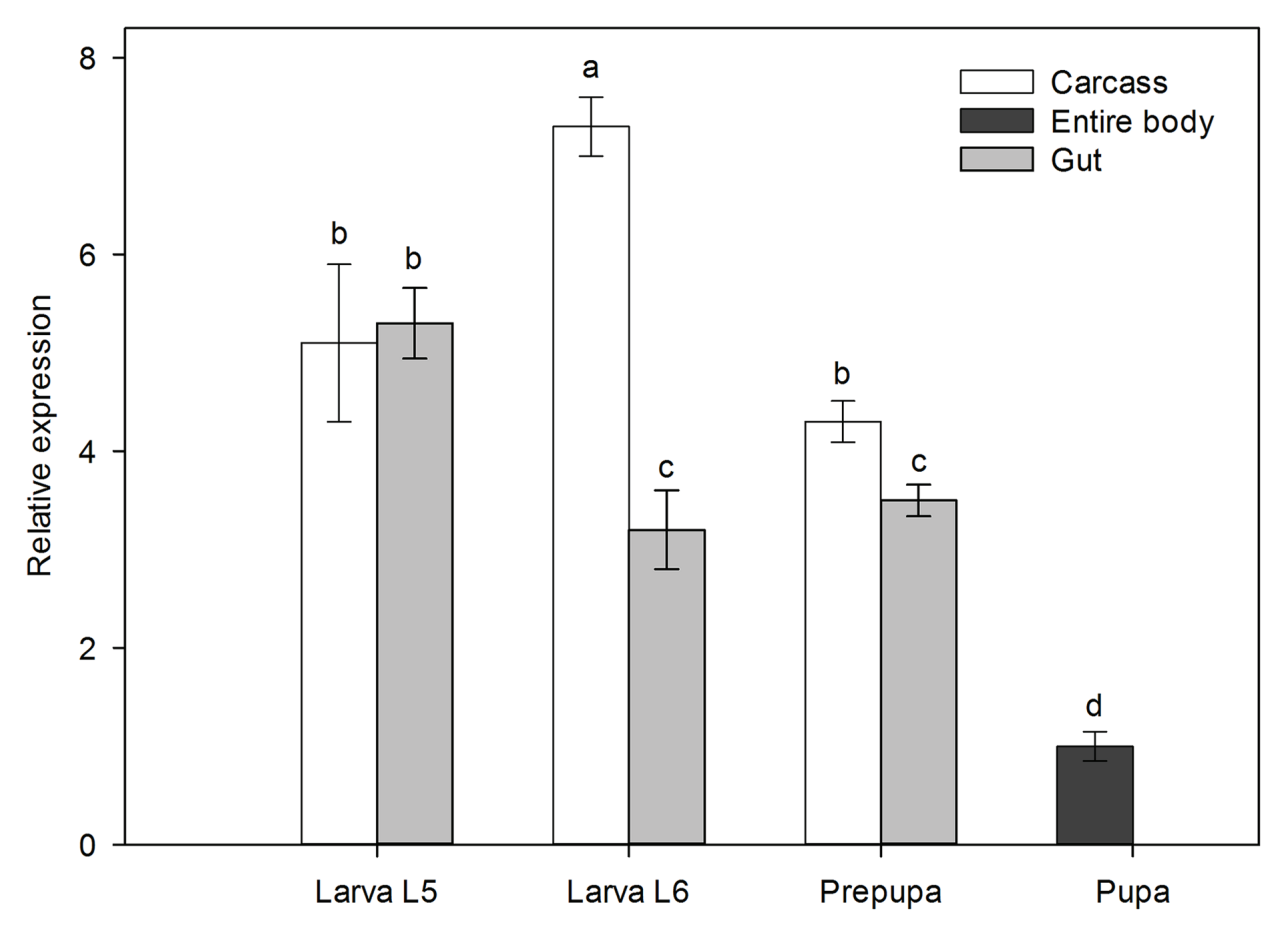
Expression of *Tljhe* (mean ± S.E) in four different late stages of sugarcane giant borer (SGB) development. Error bars were obtained from at least three independent biological replicates (five larvae per replicate). Different letters represent significant differences among different development stages (Tukey-TSD *p* < 0.05).

**Figure 5:**
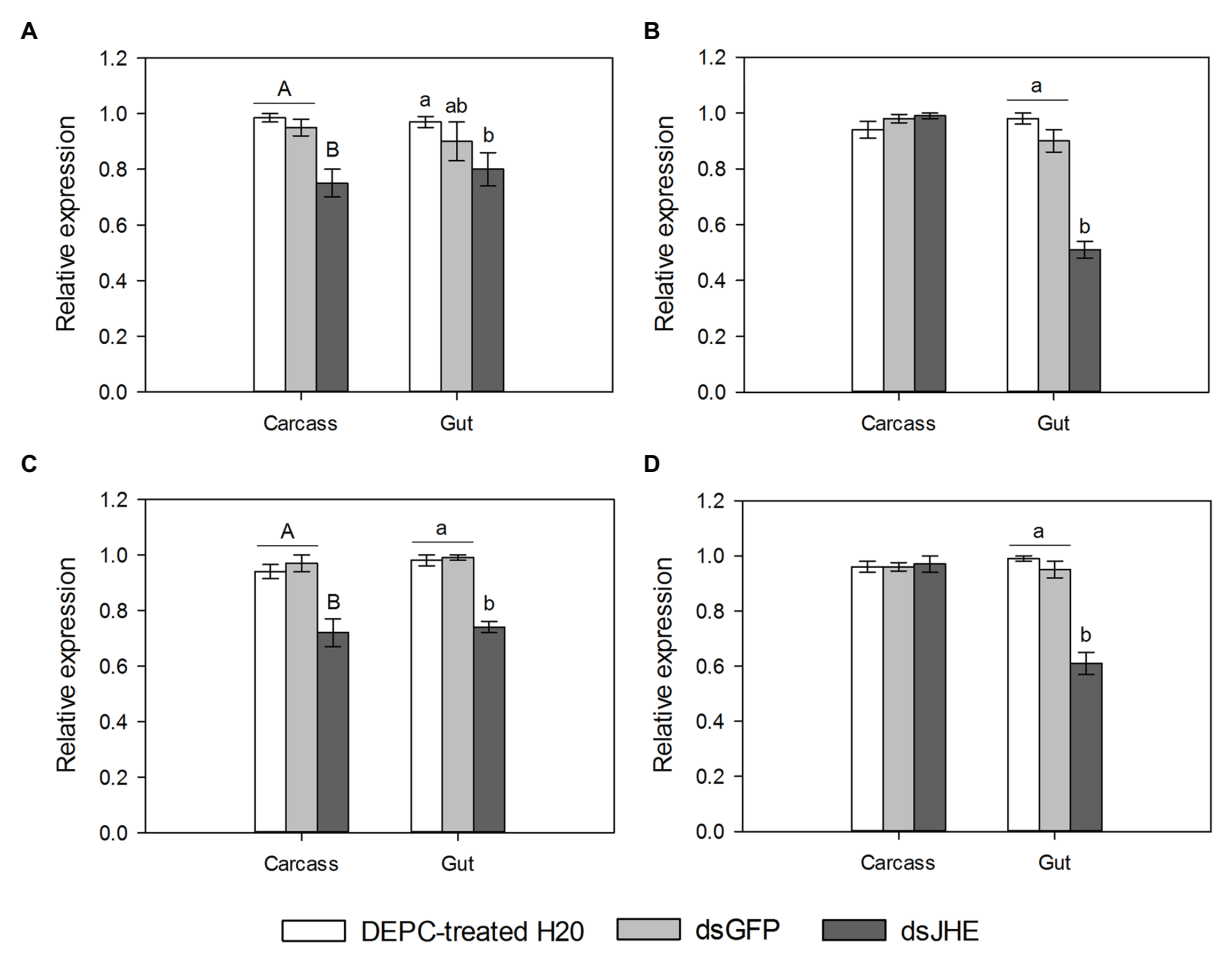
Expression of *Tljhe* (mean ± S.E) in response to the delivery of dsJHE molecules. **(A)** L5 larvae treated with microinjection; **(B)** L5 larvae treated with oral delivery; **(C)** L6 larvae treated with microinjection; and **(D)** L6 larvae treated with oral delivery. The total amount of green fluorescent protein (dsGFP) and dsJHE delivered was 10 μg. The RNA was extracted 48 h after the delivery of dsRNA. Error bars were obtained from at least three independent biological replicates (five larvae per replicate). Different uppercase letters represent significant differences between treatments in carcass tissue. Different lowercase letters represent significant differences between treatments in gut tissue (Tukey-TSD *p* < 0.05). Statistical differences between different tissues are not shown in this graphic.

According to the expression profile of *Tljhe* in late developmental stages of the SGB (Figure 4), the highest expression level was observed in the carcass of L6 larvae, while the lowest expression level was observed in pupae. In the L6 larva and prepupa stages, expression was higher in the carcass than in the gut. Moreover, the only stage without expression differences between tissues was L5 larvae. Next, it was proven that the delivery of dsJHE molecules in larvae of *T. l. licus* reduces the expression of *Tljhe* using either microinjection or oral delivery. Using the same amount of dsRNA (10 μg), knockdown of *Tljhe* was observed in the carcass and gut (1.2-fold) of microinjected L5 larvae (Figure 5A). Otherwise, in larvae of the same instar treated by oral delivery (Figure 5B), silencing was observed only in the gut (1.7-fold). Additionally, in microinjected L6 larvae (Figure 5C), a 1.3-fold reduction in gene expression was observed in the carcass and gut. However, L6 larvae treated by oral delivery (Figure 5D) presented a 1.6-fold knockdown only in the gut. Additionally, a dose-dependent response was observed in the gut of L5 larvae treated with dsRNA but not in the carcass (Supplementary Figure S6). The highest levels of knockdown were observed in the gut using 25 μg by oral delivery (6.1-fold). Additionally, the use of 50 μg of dsRNA led to unspecific knockdown caused by dsGPF (1.4-fold).

### Impact of dsJHE in SGB Development

**Figure 6:**
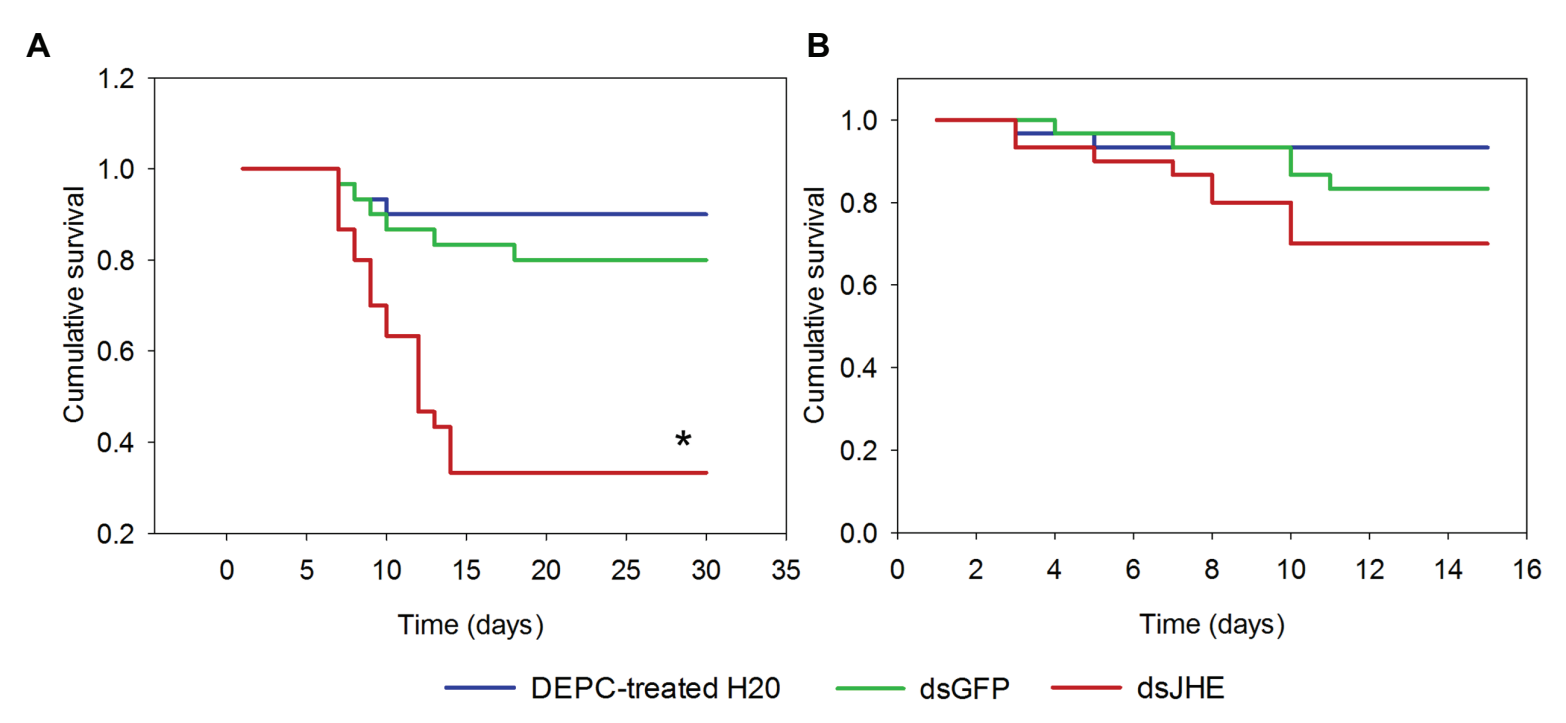
Survival curve of *T. l. licus* larvae treated with diet containing dsJHE molecules. Delivery of dsRNA was performed to different larval stages: **(A)** L5 larvae and **(B)** L6 larvae. A total amount of 100 μg was provided to each larva, divided into four applications (a single application each 48 h). Water treated with diethyl pyrocarbonate (DEPC-treated H2O) and dsGFP molecules were used as control treatments. The asterisk represents significant differences in survival between treatment with dsJHE and controls (Log-Rank test *p* ≤ 0.05, *n* = 90).

The survival distribution of L5 larvae feed on artificial diet containing dsJHE showed significant differences when compared to control treatments, dsGFP, and water treated with diethyl pyrocarbonate (DEPC-treated H2O; Mantel-Cox, *p* < 0.05; Figure 6A). After 30 days, the survival rates of individuals fed DEPC-treated water and dsGFP were 90 and 80%, respectively, while larvae treated with dsJHE showed 30% survival at the end of the experiment. Additionally, the estimation of survival in the dsJHE group was 16.9 ± 1.6 days, while DEPC-treated water and dsGFP presented an estimated survival of 27.9 ± 1.1 and 26.4 ± 1.4 days, respectively (Kaplan-Meier; p < 0.05). Otherwise, no significant differences were observed between treatments performed in L6 larvae (Figure 6B).

**Figure 7:**
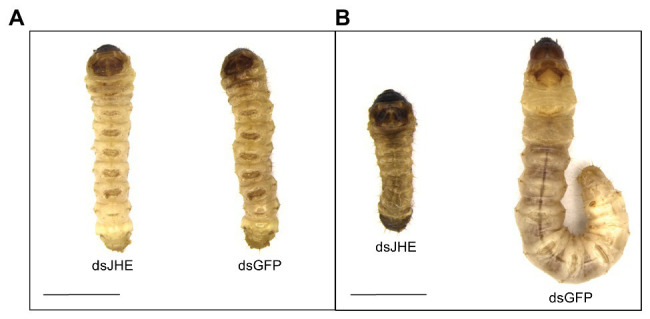
Phenotypic effect of *T. l. licus* larvae showing morphologic differences between a larva treated with dsJHE and dsGFP (control). **(A)** Larvae in T0 before delivery of the dsRNA. **(B)** Larvae after 15 days of delivery of the dsRNA. Scale bar: 5 mm. A total amount of 100 μg was provided to each larva, divided into four applications (a single application each 48 h). Photos were captured using a Leica DFC310 FX digital camera coupled to a Leica MZ12.5 Stereomicroscope.

**Figure 8:**
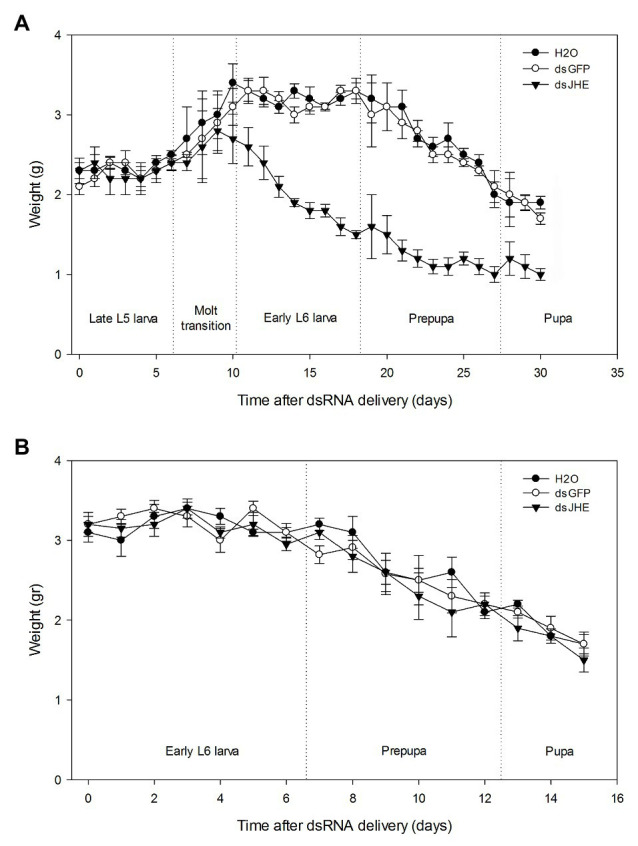
Weight of *T. l. licus* larvae over time after delivery of dsRNA molecules in artificial diet. **(A)** L5 larvae were treated. The day in which the treated larvae start losing weight (Day *Z*) were 18, 18, and 10 days for nuclease-free water, dsGFP, and dsJHE treatments, respectively. Day *z* was predicted by the generalized additive model (GAM). After day *Z*, significant differences were observed for the weight loss ratio (WLR) of dsJHE treatment (−0.08 g/day) and control treatments (−0.13 g/day for H_2_0 and −0.12 g/day for dsGFP). No differences were observed between H_2_0 and dsGFP treatments. Before Day *Z*, no differences were observed between any of the treatments, 0.06 g/day for H_2_0, 0.07 g/day for dsGFP, and 0.05 g/day for dsJHE (*F* Test, Bonferroni correction, *p* ≤ 0.05). **(B)** L6 larvae were treated. None significant day *Z* was predicted by the GAM model. None significant differences were found when comparing the WLR of all treatments (*F* Test, Bonferroni correction, *p* ≤ 0.05). The average weight and error bars (±S.T) of 90 insects per treatment are shown. A total amount of 100 μg was provided to each larva, divided into four applications (a single application each 48 h). DEPC-treated water (H_2_0) and dsGFP molecules were used as control treatments.

Additionally, signals of nutritional deficit were observed in larvae treated with dsJHE in comparison to the control (Figure 7). This fact was supported by an increased weight loss in dsJHE-treated individuals related to the control treatments (Figure 8). Based on the GAM model, the predicted day in which the larvae treated in L5 start losing weight (Day *Z*) were 18, 18, and 10 days for nuclease-free water, dsGFP, and dsJHE treatments, respectively. Otherwise, for larvae treated in L6 was not observed a significant day *Z* in any of the treatments. Then, a simple regression was performed instead of two (Supplementary Table S7). Finally, in the experiments initiated with L5 larvae, the NDI at the end of the experiment was significantly different between larvae treated with dsJHE (8.1 ± 1.6 g) and the control treatments H_2_O (14.6 ± 0.6 g) and dsGFP (14.4 ± 0.8 g; *t*-test *p* < 0.05, ±SEM). Notably, the majority of NDI reduction was recorded in the first 10 days with a 2.7-fold reduction compared with the second part (days 10–20, 1.6-fold) and the third part (days 20– 30, 1.3-fold) of the experiment (Table 1).

**Table 1:** Diet consumption values of *Telchin licus licus* larvae fed with double-stranded RNA (dsRNA) molecules.

Treatment	NDI (g)			
	0–10 days	10–20 days	20–30 days	Cumulative
H_2_O (L5)	5.0 ± 0.2 **a**	8.2 ± 0.5 **c**	1.3 ± 0.5 **d**	14.6 ± 0.7 **e**
dsGFP (L5)	5.0 ± 0.2 **a**	8.3 ± 0.4 **c**	1.7 ± 0.4 **b**	14.5 ± 0.8 **e**
**dsJHE (L5)**	1.9 ± 0.7 **b**	5.2 ± 0.8 **a**	1.1 ± 0.5 **d**	8.2 ± 1.7 **c**
H_2_O (L6)				8.3 ± 0.4 **c**
dsGFP (L6)				8.2 ± 0.3 **c**
**dsJHE (L6)**				6.3 ± 0.3 **f**

NDI, net diet intake; L5, fifth larval stage; L6, sixth larval stage. Cumulative NDIs for L5-treated and L6-treated larvae were recorded after 30 and 15 days of the experiment, respectively. Means ± SEM followed by the different letter (bold) on the line are significantly different (Tukey test, *p* < 0.05).

### Off-Target Sequences Analysis

We obtained a total of 29 significant hits from blast the *Tljhe* sequence to four model species of holometabolous insects (Supplementary Table S6). The highest identity percent was 96 with *D. ponderosae* (XM_019913361.1), and the smaller percent was 66.3 with two *B. mori* sequences. Overall, 75% of identity was observed between *Tljhe* and the sequences obtained. However, none suitable siRNA, from our designed dsRNA, was found in any of the 29 sequences evaluated. Otherwise, from 285 possible siRNAs, 148 suitable sequences were obtained for the *Tljhe* sequence (Supplementary Table S8).

## Discussion

### Transcriptome Analysis

In the previous transcriptome of the SGB, sequenced using pyrosequencing technology, 23,824 contigs were reported, of which 8,708 encoded proteins described in the NR database (de Assis Fonseca et al., 2015). In this study, using Illumina sequencing, we generated a transcriptome with approximately five times more contigs and significantly larger reads (approximately 100X). We observed a pattern common to other transcriptomes of insects challenged with different diets. The presence of DEGs related to digestion, detoxification, and transport is observed most frequently in similar studies (Roy et al., 2016; Huang et al., 2017; Birnbaum and Abbot, 2019; Cantón and Bonning, 2020). Among all major processes enriched in our analysis, transport of substances was the only one in which all related transcripts were overexpressed in one condition (sugarcane-fed larvae). Transport molecules can be associated with nutrition or with detoxification, depending on the molecule that is carried. In previous publications, it was reported that the presence of multiple transcripts encoding transporter molecules indicates a generalist approach to food sources (Govind et al., 2010; Roy et al., 2016). It is important to highlight that the insects were obtained from a population adapted to field sugarcane. Thus, the results from digital expression are expected to reflect the immediate mechanisms that larvae of the SGB use to adapt into new diets. In our study, most of the natural defenses from the host are not considered and predicted proteins observed in the expression profile are presumably related to adaptation mechanisms to exploit a new distribution of nutrients.

### Predicted Esterases in the SGB

Esterases are a diverse group of enzymes with multiple associated functions. In insects, some of them act as an important component of the xenobiotic detoxification system, while others are involved in the metabolism of hormones and the regulation of nervous signals (Oakeshott et al., 2010). The esterases hydrolyze ester groups to their component alcohols and acids. The classic classification of esterases is determined by the type of inhibitor that binds to each enzyme, being separated into four groups: acetylesterases, arylesterases, carboxylesterase, and cholinesterases (Aldridge, 1973). As expected, the number of predicted carboxylesterases found in our transcriptome was considerably higher than the number of predicted acetylcholinesterases (AChE). Because AChE is exclusively related to nerve cells, the expression of these genes in digestive tissues is uncommon. Otherwise, most carboxylesterases are associated with a wide range of functions, such as detoxification and development (Oakeshott et al., 2010). We also found five DEGs encoding carboxylesterases (Supplementary Figure S5), two of which encode secreted esterases related to JH metabolism. Thus, exposure to different diets not only regulates the expression of genes involved in the digestive metabolism of *T. l. licus* but could also alter the expression profile of development-associated genes. In other insects, this regulation resulted in different developmental times, metamorphosis delays, and variations in life history traits and established one of the most important steps in adaptation to new hosts and food sources (Li et al., 2016; Roy et al., 2016; Zhong et al., 2017).

### Knockdown of a Juvenile Hormone Esterase Gene

We focused on studying a gene encoding a JHE enzyme because of its importance for insects’ survival and development. Also, due to its specificity is a potential target for RNAi-based pest management. Usually, JHE is produced in the insect fat body, but the expression of JHE-encoding genes has been observed in other tissues, such as the gut (Zhu et al., 2018; Huang et al., 2019), as confirmed by our transcriptome. Furthermore, local activity of JH in the gut (Rahman et al., 2017) and neofunctionalization of JH isomers in nervous tissues (Steiner et al., 2017) have been recently described in *D. melanogaster*. Our findings suggest that the gene *Tljhe* has an important role in the development of *T. l. licus* larvae. Moreover, this gene is differentially induced by the diet source and influences development in a highly specific stage of the larval phase. Overall, in the late premetamorphic stages of the SGB, the expression of *Tljhe* was higher in the carcass than in the gut with the exception of larva L5, which presents the peak expression in gut tissue and equals the expression levels of the carcass (Figure 4).

The canonical function of JHE in insects is the degradation of JH in specific stages of larval development. Thus, precocious metamorphosis or incorrect molting are avoided (Kamita and Hammock, 2010). In our study, we observed different responses to the knockdown of *Tljhe*, depending on which larval instar was treated. Interestingly, even leading to similar knockdown effects when applied in L5 and L6 larvae (Figure 5), the molecules of dsJHE only cause mortality (Figure 6) and loss of weight (Figure 7) in individuals treated at the fifth larval instar. Thus, we hypothesized that the impact caused by the knockdown of *Tljhe* was correlated with the feeding behavior of the larva, which notably differs between L5 and L6 instars. Usually, the last larval instars in holometabolous insects are characterized by a decrease in the food intake rate as a preparation to enter the pupal phase (Heming, 2018). As our data show, knockdown of *Tljhe* produced an abnormal decrease in the amount of diet consumed by the larvae (Table 1), driving the nutritional deficit and death of the individuals. This effect was not lethal in larvae treated at L6 instar, presumably due to the innate physiological adaptation that the larva exhibits to starvation in the prepupa stage.

Another result to highlight is that we did not observe a systemic RNAi response in the L5 larvae treated with dsRNA. Only a slight knockdown effect was observed in the carcasses of individuals treated by oral delivery with the highest dose. However, the same individuals presented a strong knockdown of *Tljhe* in the gut tissue at all doses tested (Supplementary Figure S7). This finding is further evidence of the importance of local regulation of gene expression that controls development of the SGB by modulating its feeding behavior. Most of the studies validating the function of the *jhe* gene in insects have been performed by RNAi applied directly into the hemolymph instead of focusing on a specific tissue (Mackert et al., 2008; Kontogiannatos et al., 2013; Duan et al., 2016; Grover et al., 2019). Finally, only a small number of papers reported successful knockdown of *jhe* by oral delivery or its characterization in gut tissues, although neither of them showed the starvation-induced behavior described in our work (Wheeler et al., 2010; Chikate et al., 2016; Vatanparast et al., 2017; Grover et al., 2019).

## Conclusion

In this study, for the first time, we presented a tissue-specific transcriptome of the SBG insect pest and revealed the gene expression profiles of the gut from larvae feed on different diets. The genes highlighted in this study involving digestion, transport, and detoxification processes are potential pest management targets, as some of them could determine the success or failure to adapt to a specific host or diet. Additionally, we suggested a starvation-regulatory function of a *jhe* gene expressed in the gut that could be related to the nutritional resources available. The knockdown of the *Tljhe* gene triggered an alteration of development at a specific stage of the larval cycle. However, as JHE is a secreted enzyme, it remains to be determined whether this local knockdown can cause abnormal levels of JH in the hemolymph or if it only produces an effect in the gut cells. Isolation and quantification of JH metabolites, as well as the measurement of the enzymatic activity of JHE, are the next steps to elucidate the function and mechanism of this enzyme in the metabolism of the SGB’s gut.

## Data Availability Statement

The datasets presented in this study can be found in online repositories. The names of the repository/repositories and accession number(s) can be found at: https://dataview.ncbi.nlm.nih.gov/object/PRJNA645418?reviewer=o0d106g2ikbeiami618vqu3eh4.

## Author Contributions

DN performed the RNAi experiments. DN and FA performed the data curation, formal analysis of data, and interpretation of results. DN, FA, PG, and RT contributed to bioinformatics analysis, figure production, and statistical analysis. DN, FA, MG, FF, and JA wrote and edited the manuscript. LM, JA, and FF designed the methodology and performed the RNA-seq experiments. MG, AN, and MS supervised the research and obtained funding and resources. MG, LM, and JA contributed to conceptualization of the project. MG was the leader and administrator of the project. All authors contributed to manuscript revision, read, and approved the submitted version.

### Conflict of Interest

The authors declare that the research was conducted in the absence of any commercial or financial relationships that could be construed as a potential conflict of interest.

## Footnotes

^1^http://ewindup.info/miner/
