# Supplementary material for: Transcriptome Analysis and Knockdown of the Juvenile Hormone Esterase Gene Reveal Abnormal Feeding Behavior in the Sugarcane Giant Borer
Source: Front Physiol. 2020 Oct 28;11:588450. doi: 10.3389/fphys.2020.588450 (PMC7655874; doi:10.3389/fphys.2020.588450)
Supplement: Supplementary file 1 [file Data_Sheet_1.pdf]

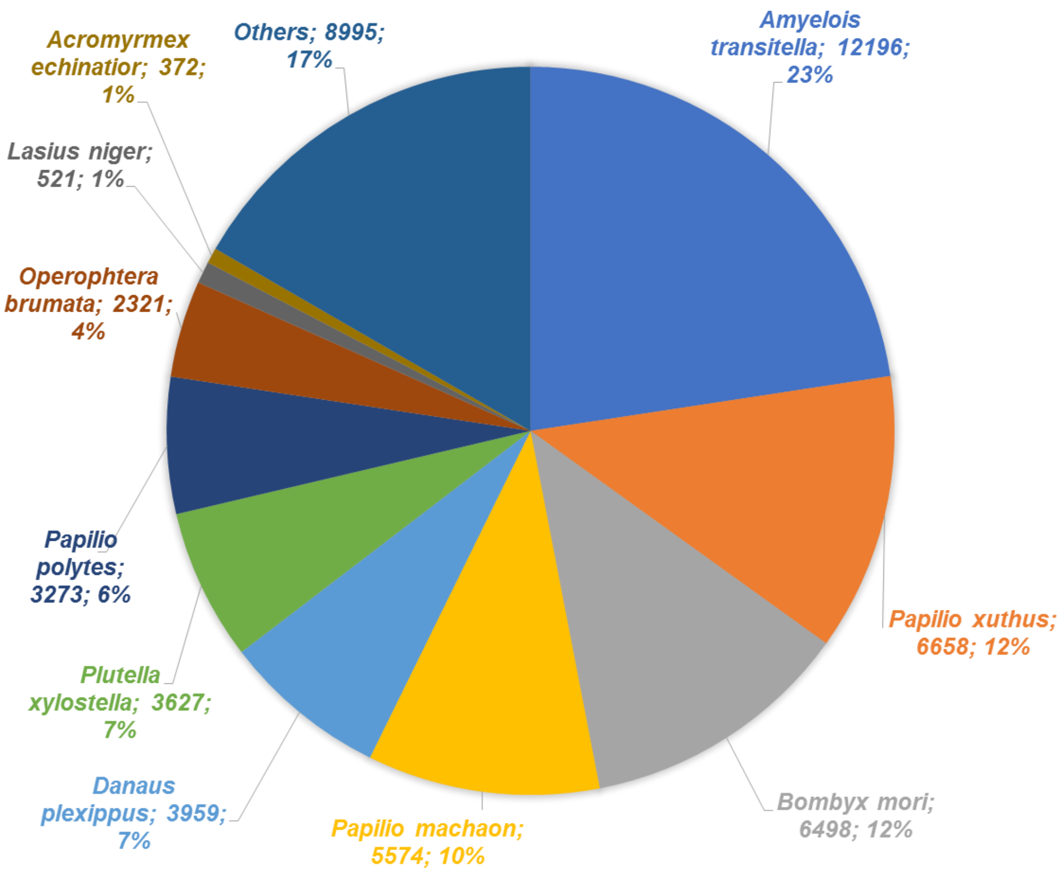

Figure S1. Distribution of top-hit species from BLAST analysis comparing the SGB transcriptome to the NR database of the NCBI. Legend shows species name; number of hits (match contigs); percent.

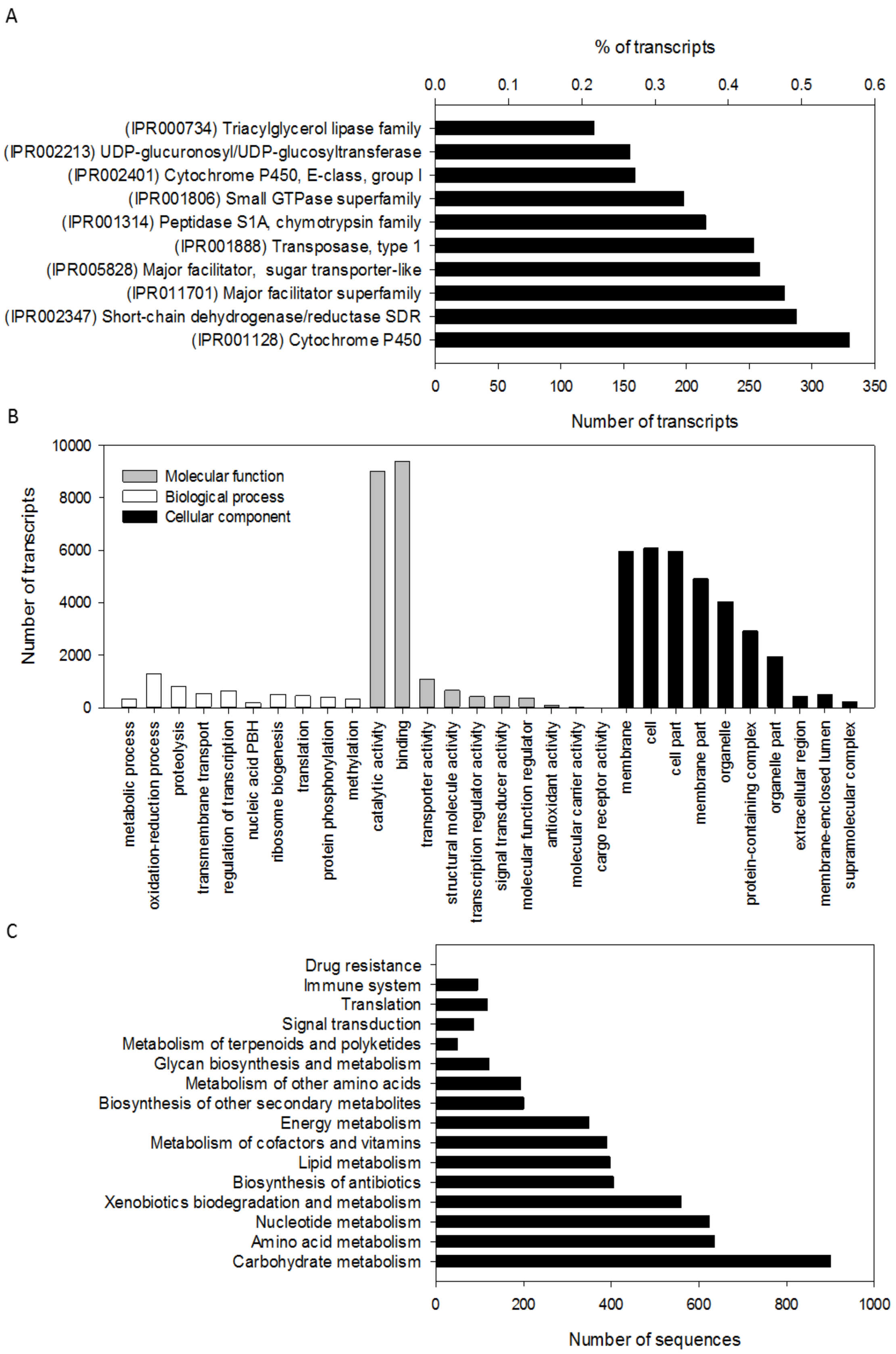

Figure S2. Annotation summary of SGB transcriptome. A) Top ten protein families (y-axis) annotated by using InterPro database. B) Gene ontology distribution of level 2 terms (x-axis). C) Distribution of metabolic pathways (y-axis) annotated with KEGG database.

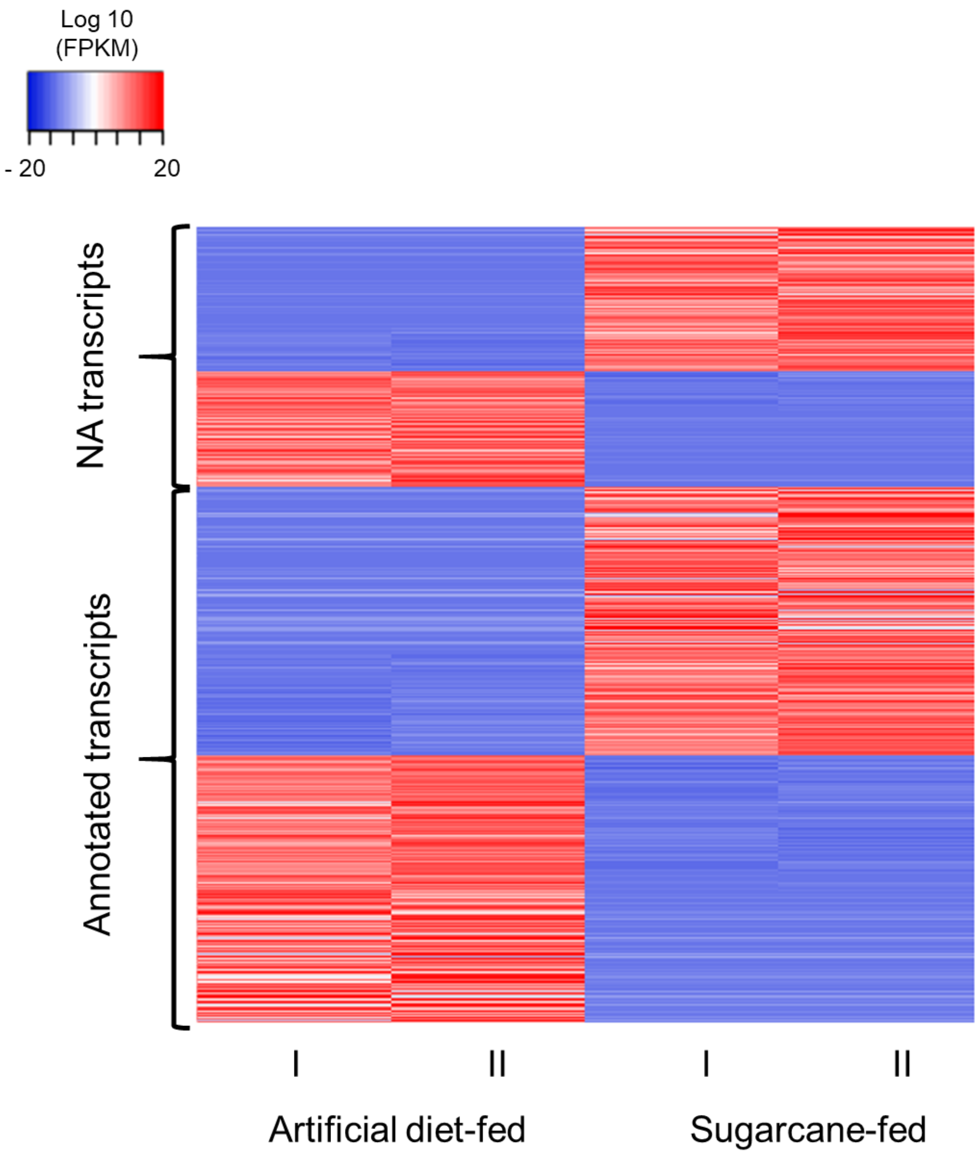

Figure S3. Heatmap showing distribution of differentially expressed genes of the SGB treated with different diet sources. Roman numerals represent different biological replicates. FPKM: fragments per kilo base per million of reads. NA: without hits in the protein NR database from the NCBI.

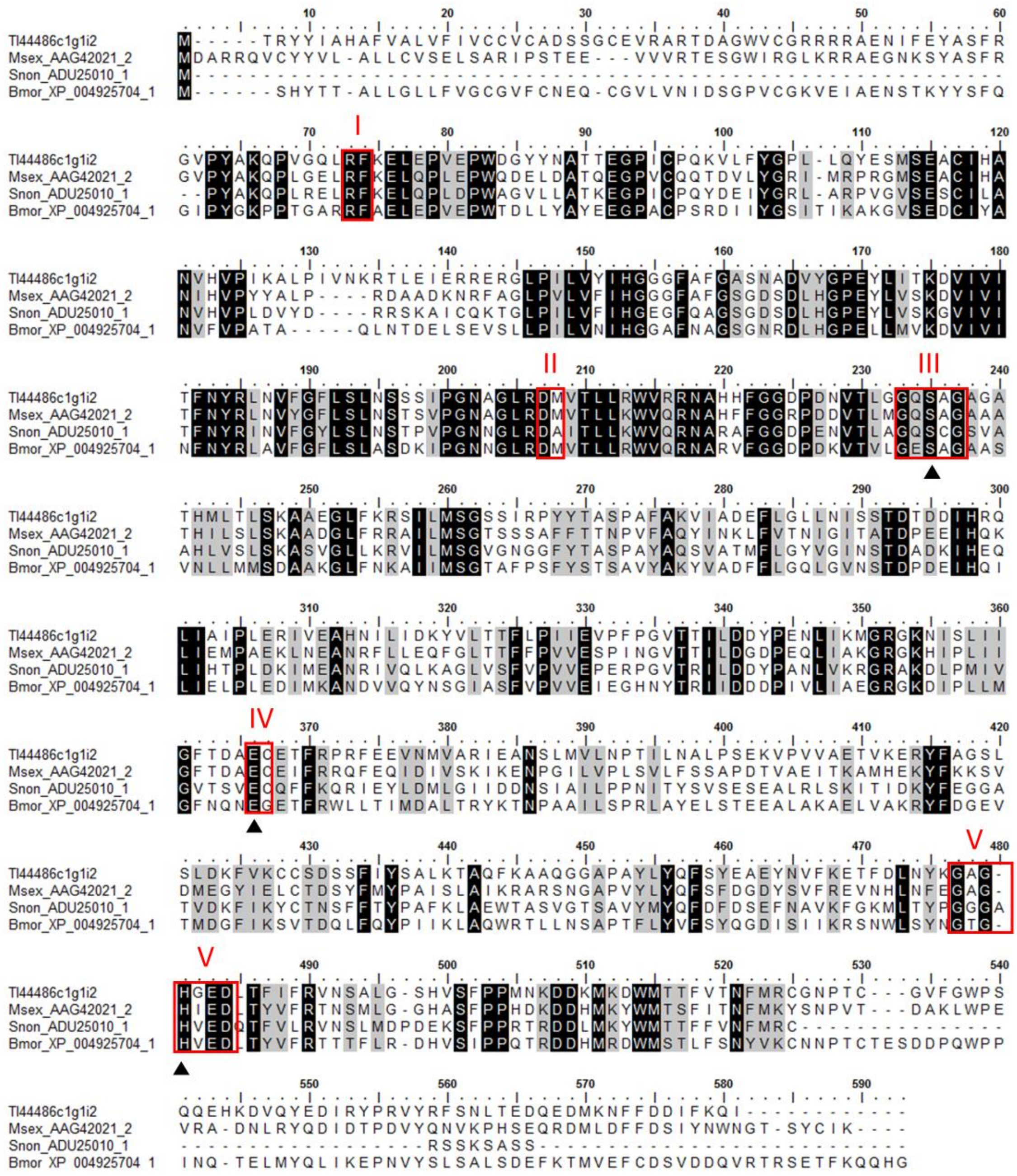

Figure S4. Amino acid sequence comparison between Tljhe (Tl44486c1g1i2) and other insect JHEs. Sequences are aligned by using the MAFFT algorithm. The major catalytic motifs conserved in all insect JHEs are within red boxes and identified by roman numerals in red. The residues that form the catalytic triad are indicated by black triangles. Species abbreviations correspond to Msex: *Manduca sexta*. *Bombyx mori*. Snon: *Sesamia nonagrioides*. Accession numbers (NCBI) are given right to the species abbreviation. Residues shaded in black and grey represent 100% and 80% conserved regions, respectively.

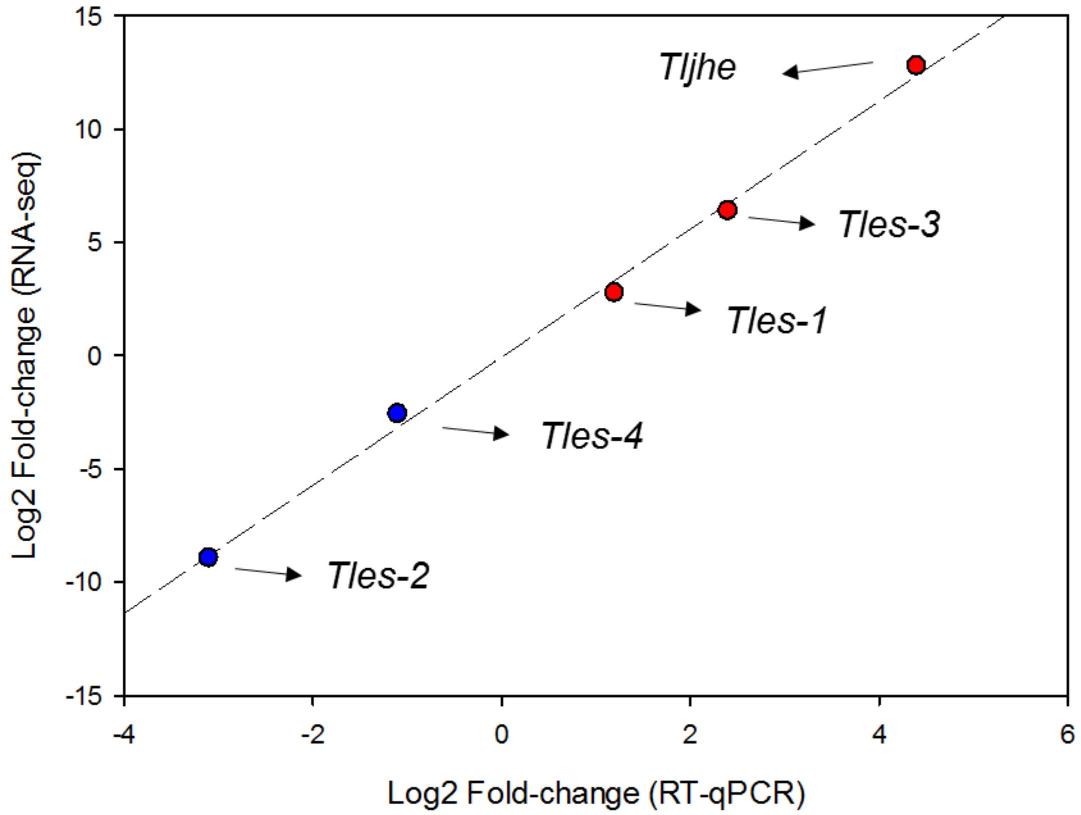

Figure S5. Correlation between expression values of DEGs encoding predicted esterases from *T. l. licus*, measured by RNA-seq and RT-qPCR. Circles represent transcripts overexpressed (red) and underexpressed (blue) in sugarcane-fed larvae compared with larvae fed on artificial diet. ***Tljhe*****:** juvenile hormone esterase. ***Tles-1*****:** esterase from contig Tlic2317c4g1i1. ***Tles-2*****:** esterase from contig Tlic44789c0g1i1.***Tles-3*****:** esterase from contig Tlic38218c1g4i5. ***Tles-4*****:** esterase from contig Tlic48599c3g1i4.

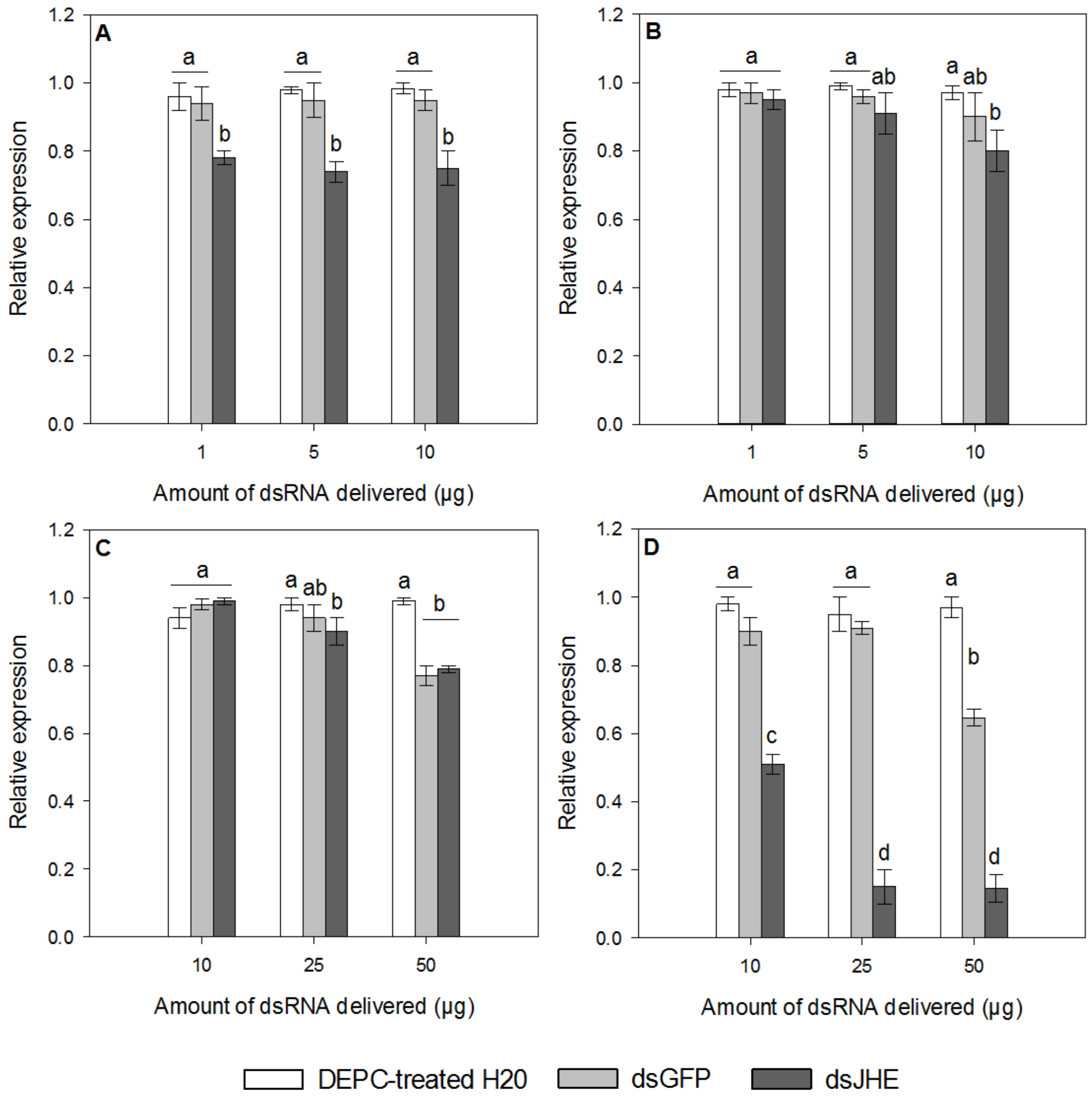

Figure S6. Expression of *Tljhe* as response to the delivery of dsJHE molecules. (**A**) Expression in carcass after microinjection (**B**) Expression in carcass after oral delivery (**C**) Expression in gut after microinjection (**D**) Expression in gut after oral delivery. Treated larvae were all at L5 instar. Error bars were obtained from at least three independent biological replicates. Different lowercase letters represent statistical differences between treatments (Tukey-TSD $p<0.05$).

Table S1. Composition of artificial diet for rearing of *T. l. licus* larvae.

COMPOUND	AMOUNT
Casein	20 g
Yeast extract	10 g
Sugar	60 g
Ascorbic acid	10 g
Vitamins solution	11 mL
Wesson salts	7.5 g
Cholesterol	300 mg
Sodium benzoate	300 mg
Ampicillin	80 mg
Nystatin	800,000 UI
Choline chloride (50%)	2 mL
Distillated-water	800 mL
Agar	25.1 g

Table S2. List of primers used for RT-qPCR. Efficiency show was calculated using Real-time PCR Miner software. **Fw**: Forward primer. **Rv**: Reverse primer. ***β-act:*** β-actin. ***α-tub:*** α-tubulin. ***gapdh:*** Glyceraldehyde 3-phosphate dehydrogenase. ***ef-1α:*** Translation elongation factor 1 alpha. ***ubq:*** ubiquitin. ***prpp:*** Phosphoribosyl pyrophosphate synthetase. ***rps18:*** ribosomal subunit 18S. ***Tljhe*****:** juvenile hormone esterase. ***Tles-1*****:** esterase from contig Tlic2317c4g1i1. ***Tles-2*****:** esterase from contig Tlic44789c0g1i1.***Tles-3*****:** esterase from contig Tlic38218c1g4i5. ***Tles-4*****:** esterase from contig Tlic48599c3g1i4.

Gene	Primer Sequence Forward (5'-3')	Amplicon (bp)	Mean Efficiency
β-act	Fw: ATGGTCGGTATGGGTCAGAA<br>Rv: ATGTCGTCCCAGTTGGTGAT	116	1.81
α-tub	Fw: ACTTGGTACTCGACCGCATC<br>Rv: ATGAGGAGGGAGGTGAAACC	115	1.88
gapdh	Fw: AAAGTAAAGGAGGCCGCTGT<br>Rv: CAGCAGCATACGAAGATGGA	116	1.91
ef-1α	Fw: AACATTGTCGTCATCGGACA<br>Rv: GGCCTCCTTCTCGAACTTCT	114	1.89
ubq	Fw: CAATGCAAGTTGTTCGATTCA<br>Rv: GGTCTGCTGCTGGTAAACCT	113	1.83
prpp	Fw: ACAGCTATCATGGTGGACGA<br>Rv: CACACCGTGGGTTGATATTG	115	1.93
rps18	Fw: ACGGTGAAAATCCAGTTTGG<br>Rv: GGACACGGATTCCCAGTAGA	118	1.92
Tljhe	Fw: TTGTTGAGTCGTCGCTTCC<br>Rv: TGAAGCCCACGATGAATGG	105	1.90
Tles-1	Fw: AGGCTGAGATGCTTGAATGG<br>Rv: GCTGCGTGGTATGGATACTG	109	1.89
Tles-2	Fw: ATCCATGGAGGTGGATTCTG<br>Rv: CCAAGTACTTCGAGTCTGTAG	118	1.88
Tles-3	Fw: CAGCGAAACAACCAATATACC<br>Rv: CAATTCATCGGCATGACACAC	114	1.89
Tles-4	Fw: TGCTGGCCTCAAAGATGTAG<br>Rv: ACCACAGCTCTCACCAAATAC	106	1.89

Table S3. Summary of reads generated by Illumina sequencing and assembly statistics.

**Library (Treatment)**	Number of sequenced reads (high-quality reads)
Sugarcane-fed I	12,322,293 (11,817,513)
Sugarcane-fed II	26,067,335 (25,024,195)
Artificial diet-fed I	14,837,428 (14,244,726)
Artificial diet-fed II	20,991,014 (20,219,338)
Reference sample	57,057,629 (42,450,109)
**Assembly statistics**	
Number of Unigenes	85,201
Number of Contigs	121,538
GC %	36.71
Completed BUSCOs	1,045 (98.0%)
Single copy BUSCOs	596 (55.9%)
Duplicated BUSCOs	449 (42.1%)
**Contigs Statistics**	Contigs
Median (bp)	643
Mean (bp)	1,190.69
Total assembled bases:	144,713,645
Contig N50 (bp)	2,014

Table S4. List of significant GO terms obtained by enrichment analysis of the DEGs found in the transcriptome of the SGB. FDR: false discovery rate value. *Active with either ribo- or deoxyribonucleic acids and producing 5'-phosphomonoesters. N: number of transcripts.

**GO Category**	**GO name**	**GO Id**	**N Up**	**N Down**	**FDR**
**Biological Process**	transmembrane transport	GO:0055085	67	0	0.00
	establishment of localization	GO:0051234	91	0	0.00
	transport	GO:0006810	91	0	0.00
	localization	GO:0051179	91	0	0.01
	DNA integration	GO:0015074	30	0	0.02
	transposition	GO:0032196	18	0	0.02
	transposition, DNA-mediated	GO:0006313	18	0	0.02
	DNA recombination	GO:0006310	19	0	0.03
	amino sugar metabolic process	GO:0006040	2	12	0.00
	aminoglycan metabolic process	GO:0006022	2	12	0.04
	chitin metabolic process	GO:0006030	2	12	0.00

	glucosamine-containing compound metabolic process	GO:1901071	2	12	0.00
	lipid metabolic process	GO:0006629	7	23	0.00
	metabolic process	GO:0008152	243	289	0.00
	oxidation-reduction process	GO:0055114	42	68	0.00
**Cellular Component**	integral component of membrane	GO:0016021	92	0	0.00
	intrinsic component of membrane	GO:0031224	92	0	0.00
	membrane part	GO:0044425	95	0	0.00
	membrane	GO:0016020	119	0	0.02
	mitochondrial outer membrane	GO:0005741	6	0	0.04
	organelle outer membrane	GO:0031968	6	0	0.04
	outer membrane	GO:0019867	6	0	0.04
	collagen trimer	GO:0005581	0	3	0.02
	extracellular region	GO:0005576	4	24	0.00
	microbody	GO:0042579	1	6	0.01
	peroxisome	GO:0005777	1	6	0.01
**Molecular Function**	transmembrane transporter activity	GO:0022857	56	0	0.00
	hydrolase activity, acting on ester bonds	GO:0016788	31	0	0.02
	active transmembrane transporter activity	GO:0022804	17	0	0.02
	endoribonuclease activity	GO:0004521	11	0	0.02
	transporter activity	GO:0005215	57	0	0.02
	RNA-DNA hybrid ribonuclease activity	GO:0004523	9	0	0.02
	hydrolase activity	GO:0016787	132	0	0.02
	endoribonuclease activity, producing 5'-phosphomonoesters	GO:0016891	10	0	0.02
	endonuclease activity*	GO:0016893	10	0	0.03
	cofactor binding	GO:0048037	43	48	0.04
	neurotransmitter transporter activity	GO:0005326	6	0	0.04
	neurotransmitter:sodium symporter activity	GO:0005328	6	0	0.04
	solute:sodium symporter activity	GO:0015370	6	0	0.04
	secondary active transmembrane transporter activity	GO:0015291	9	0	0.04
	ribonuclease activity	GO:0004540	11	0	0.04

symporter activity	GO:0015293	8	0	0.05
endonuclease activity	GO:0004519	11	0	0.05
nuclease activity	GO:0004518	14	0	0.05
catalytic activity	GO:0003824	255	271	0.00
chitin binding	GO:0008061	2	12	0.00
cofactor binding	GO:0048037	43	48	0.00
endopeptidase activity	GO:0004175	26	37	0.01
heme binding	GO:0020037	20	33	0.00
iron ion binding	GO:0005506	18	33	0.00
oxidoreductase activity	GO:0016491	45	68	0.00
oxidoreductase activity, acting on paired donors, with incorporation or reduction of molecular oxygen	GO:0016705	19	35	0.00
peptidase activity	GO:0008233	50	52	0.05
peptidase activity, acting on L-amino acid peptides	GO:0070011	49	52	0.04
protein-N-terminal asparagine amidohydrolase activity	GO:0008418	0	3	0.02
serine-type endopeptidase activity	GO:0004252	22	26	0.05
tetrapyrrole binding	GO:0046906	20	33	0.00

Table S5. Stability (M) values of candidate reference genes evaluated with geNorm. Smaller M values correspond to more stable genes.

**Reference gene**	**M value**
β-act	1.00
α-tub	0.33
gapdh	0.19
ef-1α	0.41
ubq	0.22
prpp	0.70
rps18	0.20

Table S6. Significant hits from blastn analysis using as query the DNA sequence of *Tljhe* gene identified in the transcriptome of *Telchin licus licus*.

	Accesion number	% identity	alignment length	mismatches	gap opens	evalue
Drosophila melanogaster (7 hits)	CP023337.1	83.33	54	9	0	8.41E-06
	CP023331.1	83.33	54	9	0	8.41E-06
	AE014296.5	83.33	54	9	0	8.41E-06
	NM_141064.3	83.33	54	9	0	8.41E-06
	AC010049.7	83.33	54	9	0	8.41E-06
	AC010701.3	83.33	54	9	0	8.41E-06
	AY051599.1	83.33	54	9	0	8.41E-06
Apis mellifera (4 hits)	XM_026442009.1	78.18	55	12	0	0.002
	XM_016912970.2	78.18	55	12	0	0.002
	XM_026442008.1	78.18	55	12	0	0.002
	XM_026442007.1	78.18	55	12	0	0.002
Dendroctonus ponderosae (7 hits)	XM_019913955.1	83.02	53	9	0	2.01E-06
	BT128413.1	83.02	53	9	0	2.01E-06
	XM_019900697.1	68.00	125	40	0	0.001
	XM_019900696.1	68.00	125	40	0	0.001
	XM_019898804.1	68.00	125	40	0	0.001
	BT126863.1	68.00	125	40	0	0.001
	XM_019913361.1	96.00	25	1	0	0.044
Bombyx mori (11 hits)	AK383719.1	68.98	361	108	2	3.25E-32
	AK383719.1	69.30	228	52	8	1.14E-12
	AK383719.1	68.35	158	46	2	3.71E-06
	AY489292.1	68.98	361	108	2	3.25E-32
	AY489292.1	68.86	228	53	8	4.83E-11
	AY489292.1	68.99	158	45	2	3.05E-07
	NM_001043562.1	68.98	361	108	2	3.25E-32
	NM_001043562.1	69.30	228	52	8	1.14E-12
	NM_001043562.1	68.35	158	46	2	3.71E-06
	XM_012689988.2	66.30	273	90	2	4.83E-11

XM_012689986.2	66.30	273	90	2	4.83E-11

Table S7. Summary of GAM and linear regression models for analysis of weight loss in larvae feed on dsRNA-containing diets. Linear regression model is described as Weight Mean = b_0 + b_1 * Day after initial delivery of diet. H_2O: nuclease-free water.

Treatment	GAM deviance (%)	Day Z	Regression Before day Z	Regression After day Z
L5-treated with H_2O	97,0	18	b_0: 2.23 ± 0.404 (se)<br>b_1: 0.06 ± 0.029 (se)	b_0: 5.88 ± 0.250 (se)<br>b_1: -0.13 ± 0.010 (se)
L5-treated with dsGFP	98,5	18	b_0: 2.10 ± 0.349 (se)<br>b_1: 0.07 ± 0.025 (se)	b_0: 5.67 ± 0.136 (se)<br>b_1: -0.12 ± 0.005 (se)
L5-treated with dsJHE	99.5	10	b_0: 2.16 ± 0.082 (se)<br>b_1: 0.05 ± 0.013 (se)	b_0: 3.32 ± 0.150 (se)<br>b_1: -0.08 ± 0.007 (se)
L6-treated with H_2O	67.5	x	b_0: 3.52 ± 0.211 (se)<br>b_1: -0.10 ± 0.076 (se)	
L6-treated with dsGFP	65.9	x	b_0: 3.56 ± 0.412 (se)<br>b_1: -0.11 ± 0.055 (se)	
L6-treated with dsJHE	60.0	x	b_0: 3.55 ± 0.321 (se)<br>b_1: -0.12 ± 0.096 (se)	

Table S8. Predicted number of siRNAs obtained from mapping the dsJHE molecule to the sequences with significant hits in the BLAST analysis.

Parameters	Sequence (Accesion number: GeneBank)	Non-suitable siRNAs	Suitable siRNAs
**19-lenght siRNA**			
**0 mismatches**		0	0
	Tljhe(Control)	285	139
**1 mismatches**	AK383719.1	3	0
	AY489292.1	3	0
	NM_001043562.1	3	0
	CP023337.1	2	0
	AE014296.5	2	0
	CP023331.1	2	0
	Tljhe(Control)	285	0
**2 mismatches**	AE014296.5	83	0
	CP023331.1	60	0
	CP023337.1	59	0
	AK383719.1	10	0
	AY489292.1	10	0

	NM_001043562.1	10	0
	Tljhe(Control)	285	0
**20-lenght siRNA**			
**0 mismatches**		0	0
	Tljhe(Control)	284	140
**1 mismatches**	AK383719.1	2	0
	AY489292.1	2	0
	NM_001043562.1	2	0
	Tljhe(Control)	284	0
**2 mismatches**	AE014296.5	32	0
	CP023337.1	20	0
	CP023331.1	20	0
	AK383719.1	8	0
	AY489292.1	8	0
	NM_001043562.1	8	0
	Tljhe(Control)	284	0
**21-lenght siRNA**			
**0 mismatches**		0	0
	Tljhe(Control)	283	148
**1 mismatches**	AK383719.1	1	0
	AY489292.1	1	0
	NM_001043562.1	1	0
	Tljhe(Control)	283	0
**2 mismatches**	AE014296.5	11	0
	AK383719.1	6	0
	AY489292.1	6	0
	NM_001043562.1	6	0
	CP023337.1	4	0
	CP023331.1	4	0
	Tljhe(Control)	283	0
